# Fournier’s Gangrene Mimicking Acute Epididymo-Orchitis in an Undiagnosed Diabetic Patient

**DOI:** 10.7759/cureus.32588

**Published:** 2022-12-16

**Authors:** Antony Soares Dionísio, Joana Duarte, Maria Leonor Guia Lopes, Cristiana Camacho

**Affiliations:** 1 Internal Medicine Department, Hospital São Francisco Xavier - Centro Hospitalar de Lisboa Ocidental, Lisboa, PRT; 2 Endocrinology Department, Hospital Egas Moniz - Centro Hospitalar de Lisboa Ocidental, Lisboa, PRT

**Keywords:** septic shock, diabetes mellitus, necrotizing, fasciitis, fournier gangrene

## Abstract

Fournier’s gangrene (FG) is an infectious disease characterized by necrotizing fasciitis of the perineal, perianal, or genital area associated with aging, male gender, diabetes mellitus (DM), alcoholism, trauma, and immunosuppression states. It can rapidly evolve into sepsis, septic shock, and multiorgan failure with a high mortality rate. We present the case of a 55-year-old man who developed a severe FG, initially assumed as an epididymo-orchitis with new-onset DM. The early identification and treatment resulted in a favorable outcome, being discharged from the hospital after 21 days. Diabetic patients are more susceptible to having severe infections such as FG, hence the importance of adequate metabolic control and increased suspicion to prevent fatal complications.

## Introduction

Fournier’s gangrene (FG) was first described in 1883 by Jean-Alfred Fournier, with a series of five young males presenting with fulminant gangrene of the scrotum and penis with an abrupt onset and unknown etiology [1]. FG is defined as an infectious disease characterized by necrotizing fasciitis of the perineal, perianal, or genital area [2]. It is an uncommon condition, accounting for 0.02% of hospitalizations, although it presents an increasing incidence due to aging and a higher prevalence of diabetes mellitus (DM). This disease primarily affects men over the age of 50, with major risk factors identified such as diabetes, alcoholism, human immunodeficiency virus, lymphoproliferative diseases, chronic steroid abuse, cytotoxic drugs, and genital or perineal trauma [1,3]. Initially, it was thought that FG was a soft tissue infection caused solely by streptococcal species, but recent research indicates that it may be caused by polymicrobial infections [4]. The infection progresses as an obliterative endarteritis with micro thrombosis of cutaneous and subcutaneous arterioles resulting in the spreading of microbiological pathogens and gangrene of the surrounding tissue [1]. Diagnosis should be suspected in patients with erythema and swelling of the genitalia and perineal zone associated with disproportionate pain and subcutaneous emphysema [5]. FG can rapidly evolve into sepsis, septic shock, and multiorgan failure with a mortality estimated from 3% to 67% even with adequate early treatment. As a result, an imagiologic evaluation should be performed as soon as possible to confirm the diagnosis [3,6]. Even if some research suggests that bedside ultrasonography can be used to diagnose FG, computerized tomography remains the preferred option for locating the source of infection and its spread. Although magnetic resonance imaging provides more soft tissue detail, its limited availability in many hospitals and extended scan time have limited its use [1]. The initial measures should include fluid resuscitation, microbiological cultures, wide-spectrum antibiotic, and surgical exploration and debridement [7,8]. Patients with uncontrolled DM and persistent hyperglycemia are more likely to develop FG due to the detrimental effect of hyperglycemia in the host immune system. Furthermore atypical microorganisms such as *Candida albicans* are frequently identified and are usually associated with a higher rate of complications and a worse prognosis [1,9].

We describe a case of a 55-year-old man who had undiagnosed diabetes mellitus and developed severe FG that was initially misdiagnosed as epididymo-orchitis.

## Case presentation

**Figure 1:**
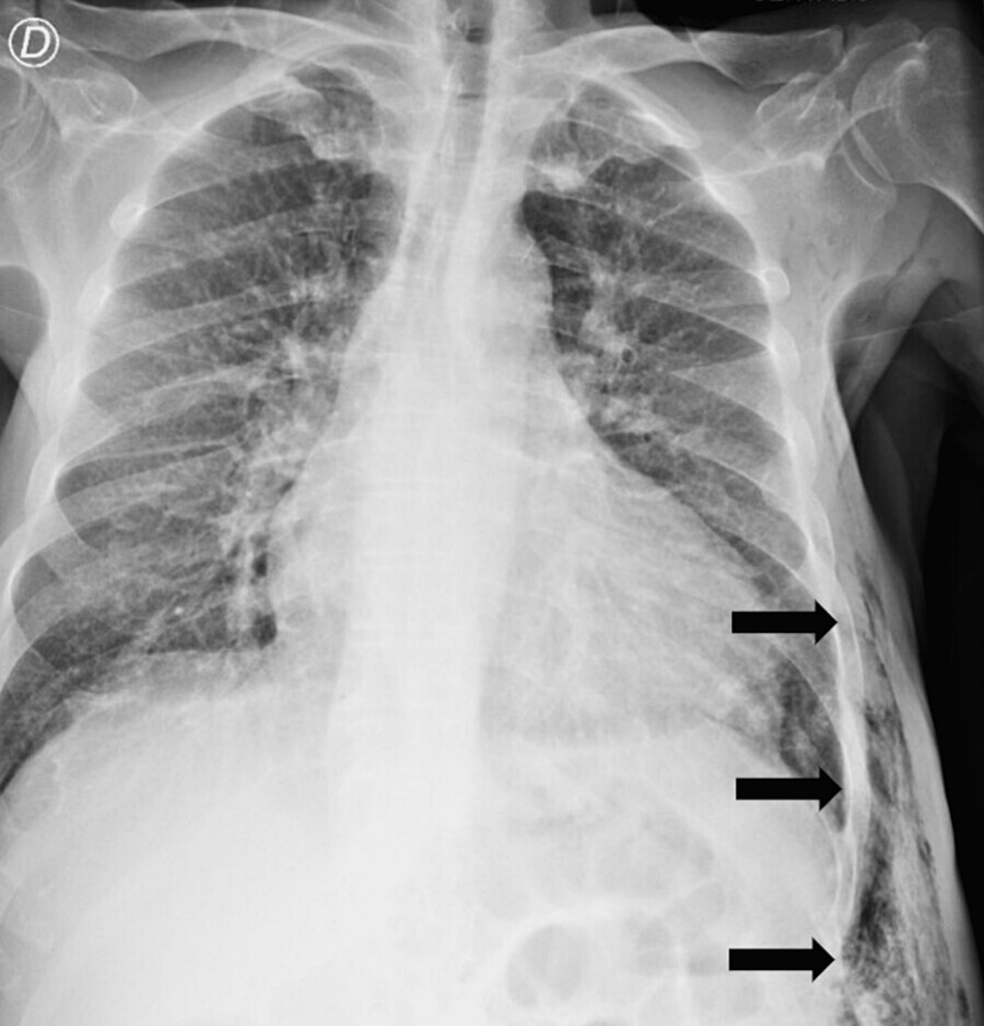
Thoracic radiography: arrows highlighting subcutaneous emphysema in the left side of the thorax and abdomen

**Figure 2:**
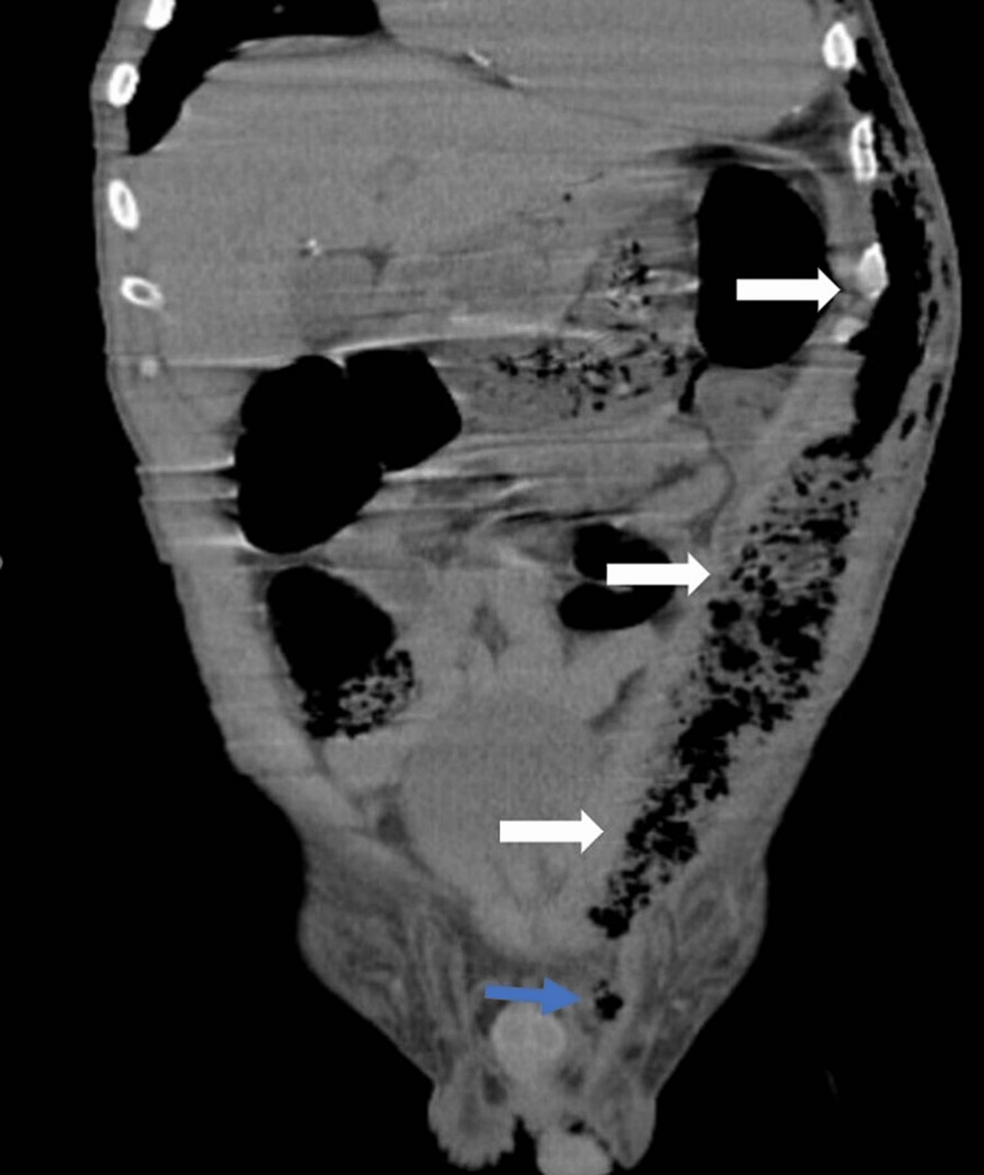
Coronal slice of thoraco-abdominopelvic CT: white arrows highlighting subcutaneous emphysema in the thoracic wall; blue arrow highlighting perineal abscess.

A 55-year-old male with a history of essential hypertension, dyslipidemia, smoking, and intermittent claudication presented to the emergency department with a four-day history of painful scrotal tenderness and fever. Concomitantly, he complained of polyuria, polydipsia, asthenia, and involuntary weight loss of 20kg (85 to 65 kg) in the last two months. Following a scrotal ultrasound, he was initially diagnosed with epididymal-orchitis and was empirically treated with amoxicillin-clavulanate. Before discharge, an evaluation by internal medicine was required due to hyperglycemia (378 mg/dl) in a patient without a previous medical history of DM. Physical examination revealed blood pressure of 107/56 mmHg, tympanic temperature of 37.7 degrees Celsius, polypnea, pallor, painful testicular edema, and erythema. On palpation, he had crepitus in the left inguinal zone that extended to the abdomen, thorax, and left axillary region. Laboratory data revealed a white cell count of 18,400/ul (normal range 4500/ul to 11,000/ul) with 17,400/ul neutrophils (normal range 2000/ul to 7500/ul) and a C-reactive protein of 27.9 mg/dl (normal range inferior to 0.5 mg/dl). Chest radiography (Figure 1) demonstrated subcutaneous emphysema in the left side of the thorax and abdomen. A thoracic-abdominopelvic computerized tomography (CT) (Figure 2) revealed a perineal and abdominal wall abscess with extension from the left hypochondrium to the upper part of the left thigh (17x4cm) with subcutaneous emphysema in the thoracic wall, confirming the suspected diagnosis of Fournier’s gangrene. The patient was immediately taken to the operating room for surgical drainage of the perineal abscess and debridement of necrotized tissue. Piperacillin-tazobactam plus clindamycin were initiated empirically. In the immediate postoperative period, the patient evolved with hypotension and lactic acidosis and was then admitted to the intensive care unit (ICU) due to septic shock. The microbiological report revealed *Actinotignum schaalii* and *Anaerococcus vaginalis*, both sensitive to penicillin, but due to the poor clinical evolution, the previous antibiotic regimen was maintained for seven days after surgery. Since the patient presented typical symptoms upon admission and the elevated glycemia levels persisted throughout the hospital stay, the diagnosis of DM was made. His result of glycated hemoglobin test (HbA1c) was 11.9%, which indicates uncontrolled glycemia. In order to achieve optimal metabolic control, the patient first required endovenous insulin. Once the patient's condition had stabilized, glargine insulin was introduced. He presented gradual improvement and resolution of septic shock, being discharged from the hospital after 21 days with a follow-up by the surgical team and consultation in endocrinology.

## Discussion

FG is a surgical emergency that has a high mortality rate. However, early detection of this condition and prompt surgical treatment can result in better outcomes, with hospital mortality reduced by 10 to 20% [6]. Its clinical manifestations range from gradual onset and evolution to abrupt onset and fulminant evolution [9]. Because streptococcal and staphylococcus species, clostridium, bacteroides, and gram-negative bacteria are the most common microbiological agents involved, treatment with a broad-spectrum antibiotic should be initiated [10].

We present the case of an undiagnosed diabetic patient with severe FG, which was initially misdiagnosed as epididymo-orchitis. The presence of exuberant subcutaneous emphysema and intense pain led to the diagnosis of FG, which was confirmed by a thoraco-abdominopelvic CT. Even though he needed to be admitted to the ICU due to hemodynamic instability in the post-surgical period, the early detection and treatment resulted in a favorable outcome. Our patient's risk factor for FG was undiagnosed diabetes, which was confirmed by the HbA1c value. Diabetic patients are at a higher risk of soft tissue and urinary infections from both typical and atypical agents, emphasizing the importance of proper metabolic control and the need for extra care in this population. On the other hand, uncontrolled glycemic values lead to metabolic dysregulation in infectious diseases [11]. Uncommon microbiological agents were discovered in our patient, which could be linked to uncontrolled diabetes. *Actinotignum schaalii* is a facultative anaerobic gram-positive rod that primarily causes urinary tract infections in elderly men and young children, and its prevalence is likely understated due to its fastidious growth [12]. *Anaerococcus vaginalis* is a gram-positive anaerobic coccus that causes ovarian, peritoneal, sacral, digital, and cervical abscesses [13].

## Conclusions

FG is an infectious disease with elevated mortality and morbidity, mainly if appropriate measures are not implemented promptly. This clinical case aims to increase awareness of the importance of a thorough physical examination and the need to consider less common diagnoses in order to treat these patients adequately to improve outcomes. Atypical microorganisms were identified, which were more likely related to uncontrolled diabetes mellitus. Diabetic patients are more susceptible to having a more severe FG, emphasizing the importance of proper metabolic control to avoid complications.
